# Protective Effect of Syringic Acid Against Cadmium-Induced Testicular Toxicity in Mice

**DOI:** 10.1007/s12011-025-04727-6

**Published:** 2025-06-25

**Authors:** Yaşar Şahin, Mehmet Eray Alçiğir, Ebru Yildirim, Hatice Kübra Nur Boran, Seydi Ali Peker, Ömer Varişli, Hüsamettin Ekici, Merve Bişkin Türkmen

**Affiliations:** 1 Department of Pharmacology and Toxicology, Faculty of Veterinary Medicine, Kırıkkale University, 71450 Kırıkkale, Türkiye; 2 Department of Pathology, Faculty of Veterinary Medicine, Kırıkkale University, Kırıkkale, Türkiye; 3 Department of Reproduction and Artificial Insemination, Faculty of Veterinary Medicine, Kırıkkale University, Kırıkkale, Türkiye; 4 Department of Biochemistry, Kırıkkale Yuksek Ihtisas Hospital, Kırıkkale, Türkiye

**Keywords:** Cadmium, INOS, Immunohistochemical, Syringic acid, Testicular toxicity, Testosterone

## Abstract

This study examined the effects of syringic acid (SA) against testicular toxicity induced by cadmium (CAD). In the study, once daily for 7 days, the control and CAD groups were administered sterile distilled water, while the CAD + SA and SA groups were administered 25 mg/kg SA. Additionally, on the first day of the study, saline solution was administered intraperitoneally to the control and SA groups, and 1.5 mg/kg CdCl_2_ was administered intraperitoneally to the CAD and CAD + SA groups. In blood, serum aspartate transaminase (AST) and alanine aminotransferase (ALT) enzyme activity, testosterone, creatine, and urea levels were evaluated. Spermatological parameters, including sperm motility, sperm viability, sperm concentration, and abnormal sperm, were evaluated. Histopathological examination and immunohistochemical analysis (inducible nitric oxide synthase (iNOS), cyclin D1, cannabinoid receptor1 (CB1)) were performed on testicular tissue. The testosterone value of the CAD group was significantly lower compared to the control and SA groups (*p* < 0.0083). In the CAD + SA group, sperm motility, sperm vitality, and sperm concentration were significantly increased compared to the CAD group. In contrast, the percentages of abnormal sperm (head and tail) were significantly decreased in the CAD + SA group compared to the CAD group (*p* < 0.0083). The rate of iNOS positivity in the SA and CAD + SA groups was similar to those in the control group. However, iNOS positivity was significantly higher in the testicles of the CAD group compared to the remaining groups (*p* < 0.0083). In conclusion, SA can be used as a preventative agent against testicular toxicity caused by CAD.

## Introduction

Nowadays, humans and animals are more commonly exposed to cadmium (CAD) from industrial sources, such as television screens, cosmetics, and batteries, natural sources; e.g., water and air (cigarette smoke, etc.), and agricultural sources, including rice and leafy vegetables [1, 2]. This non-essential heavy metal accumulates not only in human and animal bodies but also in plants [3, 5]. Due to its long half-life (about 25–30 years) and accumulation in different organs such as the testis, liver, and kidney, CAD can lead to a range of diseases and dysfunction of organs [3, 5]. Especially through its effects on the testis, it significantly impairs the fertility of humans and animals causing subfertility/infertility due to structural damage [2]. Previous studies showed that CAD caused structural disorders in the blood-testicular barrier, seminiferous tubules (degeneration), and Sertoli cells [2, 6, 8]. It also results in the loss of Sertoli and Leydig cells and/or increases the number of damaged cells [6, 9]. Oxidative stress, which occurs when the body’s cellular antioxidant defense balance is disrupted by the production of reactive oxygen species (ROS) in the body, causes various health problems, including infertility (decreased sperm quality (decreased mobility and vitality, abnormal sperm increase, etc.)). It is stated that antioxidants have important effects against ROS-induced infertility in men, and comprehensive studies need to be conducted [10, 11]. Syringic acid (SA), a phenolic compound found in vegetables, fruits, mushrooms, and grains (wheat, etc.), has anti-inflammatory, antioxidant, and antimicrobial properties [12, 14]. In addition, it has various effects, such as reducing protein carbonylation and lipid peroxidation, and shows anti-adipogenic, anti-cancer, and anti-endotoxic activity [15, 18].

Globally, the fertility rate of men, caused by environmental pollutants such as CAD, is decreasing, and there is a growing incidence of subfertility/infertility [2]. Researchers are looking for solutions to this global problem [19, 20]. Therefore, in this study, we investigated the protective effect of SA against testicular toxicity caused by CAD.

## Materials and Methods

### Animals and Experimental Protocol

**Fig. 1:**
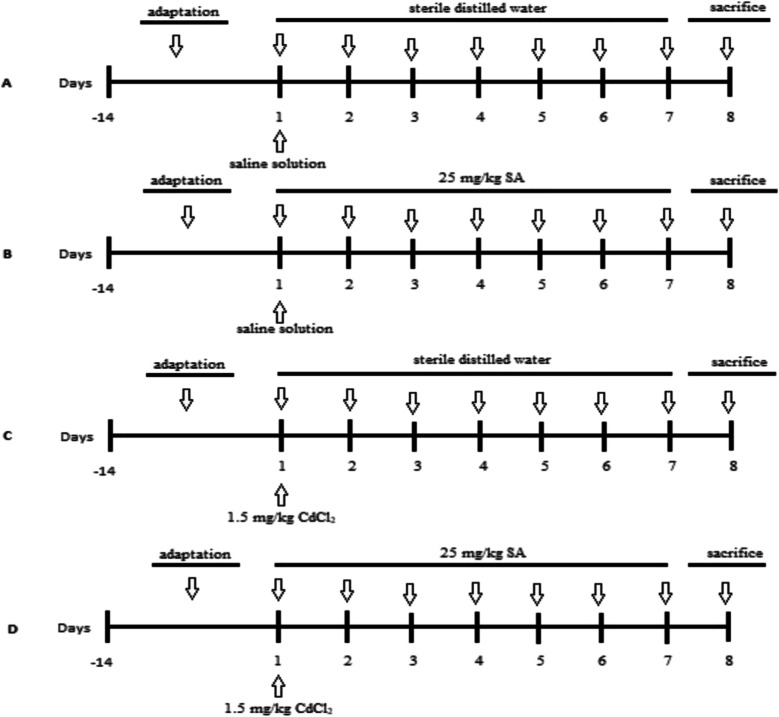
Experimental animal models. **A** Control group, **B** SA group, **C** CAD group, and **D** CAD + SA group

The study was started after receiving approval from the Animal Experiments Local Ethics Committee of Kırıkkale University (decision number: 2021–43). A total of 24 BALB/C mice (male, 6 to 8 weeks old, 30 ± 5 g), six in each group, were used in the study. On the first day of the study, saline solution was administered intraperitoneally to the control and SA groups, while 1.5 mg/kg CdCl_2_ (0.92 Cd/kg, Sigma-Aldrich, Germany) was administered intraperitoneally to the CAD and CAD + SA groups [9]. Additionally, for 7 days (single dose per day), control and CAD groups were administered sterile distilled water orally (gavage), while SA and CAD + SA groups were administered 25 mg/kg of SA (Cayman, USA) orally (gavage) [21]. The experimental animal models of the groups are shown in Fig. 1. On the eighth day of the study, mice were anesthetized by intraperitoneal administration of xylazine (10 mg/kg, Xylazinbio 2%®, Bioveta, Czech Republic) and ketamine (90 mg/kg, Vetaketam®, Vetagro, Poland). Anesthetized mice were sacrificed by blood collection from the vena cava caudalis. Also, the right and left testicles of each sacrificed animal were taken.

### Biochemical Analysis

The blood samples were collected in gel and clot activator biochemistry tubes. The blood was separated into serum by centrifugation at 3000 rpm (10 min at + 4 °C). In these samples, testosterone (measuring range, 0.025–15.0 ng/ml) levels (Cobas e 601, Roche Diagnostics, Germany), aspartate transaminase (AST) (measuring range, 3–1000 U/l), and alanine transaminase (ALT) (measuring range, 3–500 U/l) enzyme activities, creatinine (measurement range, 0.06–25 mg/dl), and urea (measurement range, 5–300 mg/dl) values (Beckman Coulter AU5800, USA) were measured.

### Sperm Collection and Cauda Epididymides Weighing

The sacrificed mice were placed in a culture dish (35 mm) containing 200 µl of Dulbecco’s phosphate buffer (D-PBS, Sigma, Germany) solution supplemented with 3 mg/ml bovine serum albumin (fraction V, Sigma, Germany), and the cauda epididymides were removed. The cauda epididymides were dissected with fine scissors to allow sperm to swim out (22 °C, 10–15 min). The sperm suspension was placed in 5-ml tubes using pipettes [22].

### Motility Analyses

The percentage of sperm motility was subjectively assessed using a phase-contrast microscope (Lecia, Germany) with a heated stage at 37 °C. For this purpose, 5 µl of sperm sample from each experimental group was put on a microscope slide and covered with a coverslip. Motile spermatozoa were then assessed on four non-overlapping fields at 40 × objective (Lecia, Germany) [22].

### Eosin-Nigrosine Staining (Viability)

Fifty microliters of dye and the same volume of semen were mixed on the slide on the heating plate (at 37 °C). After waiting for 1 min, a smear of this mixture was placed on a second slide and dried on the heating table. The dried sample was examined under a light microscope with a 40 × objective (Leica, Germany). Dead spermatozoa were detected as red due to impaired membrane integrity, while live spermatozoa were white because they had not received the dye [23].

### Abnormal Sperm Rate

The fluid fixation method was used to determine the shape and ratio of abnormal spermatozoa. Five microliters of native semen samples obtained from the epididymis was placed in a 100-µl tube containing the Hancock solution and stored in the refrigerator until analysis. Five microliters of semen was placed on the slide and covered with a coverslip. Then, 200 spermatozoa were evaluated by counting them under a microscope (Leica, Germany, 100 × objective) [22].

### Sperm Morphology Method

To determine normal and abnormal sperm morphology, trypan blue stain was used. Ten microliters of semen samples was mixed with 10 µl of trypan blue solution (Sigma, Germany). Then, 3 µl of Hancock solution was added to this mixture. Normal sperm, head-abnormal sperm, neck-abnormal sperm, tail-abnormal sperm, and cytoplasmic droplet sperm were examined under a light microscope (Leica, Germany), and images (40 × objective) were taken [22, 24].

### Histopathological Evaluation

Both testicles were sampled from the mice in all the groups. All the tissue samples were fixed in 10% buffered formalin for 48 h. After the formalin fixation, the tissues were trimmed and placed into a plastic cassette. Then, the samples were treated with graded ethanol and xylol series (Leica, Germany) and embedded in paraffin molds (Thermo, Germany). Sections of 6 µm thickness were cut using a rotary microtome (Shandon, UK) and stained according to the hematoxylin–eosin (H&E) staining procedure [25]. These sections were placed under a digital optical light microscope and images were taken with a camera attachment (Olympus, Japan).

### Immunohistochemical Analysis

The streptavidin–biotin peroxidase (Strept ABC) method was applied to the silanized slides according to the manual instruction of in the peroxidase detection system (Novocastra RE7120-CE, Leica, Germany). Rabbit polyclonal primary antibodies (Table 1) were dropped onto the slides and incubated at 37 °C overnight. For the control section, phosphate-buffered saline (PBS) was dropped into the sections for negative controls as primary antibodies. As a positive control, selected antibodies were incubated with mice tissue sections. The sections reacted positively to show cross-reaction. Then, 3,3′-diaminobenzidine (DAB) chromogen was used for color reaction. For counter-stain, the sections were stained with Gill’s hematoxylin. Finally, the sections were mounted using coverslip and Entellan (Merck, Germany). Necessary clarifications have been given in Table 1.

**Table 1:** Markers used in immunohistochemical examination

Marker name	Trademark-catolog number	Dilution
iNOS	Abcam, ab3523	1/200
Cyclin D1	Antibodiesonline, ABIN782606	1/200
CB1	NovusBio, NLS32	1/200

*iNOS*, inducible nitric oxide synthase; *CB1*, cannabinoid receptor 1

### Lesion Scoring and Statistical Analysis

Data obtained from the study were given as arithmetic mean ± standard error. SPSS software (PASW, ver. 18.0. Chicago, USA) was used for statistical analyses. One-way analysis of variance (ANOVA) was performed, which showed a parametric distribution of biochemical results (ALT and creatinine), and a post hoc analysis of the Duncan test was undertaken to check the significance between the groups (*p* < 0.05). The Kruskal–Wallis test was used for the parameters showing a non-parametric distribution (testosterone, AST, and urea), and the Mann–Whitney *U* test (Bonferroni correction, *p* < 0.0083) to determine the significance between the groups. Some sperm parameters, including sperm viability, sperm concentration, and cytoplasmic droplet sperm, showed a parametric distribution. Therefore, one-way ANOVA and the post hoc Tukey test were performed for their evaluation (*p* < 0.0001). Other sperm parameters, including sperm motility, right testicular weight, left testicular weight, head abnormal sperm, neck abnormal sperm, and tail abnormal sperm, showed non-parametric distribution and were analyzed using the Kruskal–Wallis test and the post hoc Mann–Whitney *U* test (Bonferroni correction, *p* < 0.0083). The Kruskal–Wallis test was used for the immunohistochemical findings showing a non-parametric distribution. The Mann–Whitney *U* test (Bonferroni correction, *p* < 0.0083) was used to determine the significance between the groups. Histopathological findings were semiquantitatively scored (10 high-power fields or HPFs: (−), negative 0%; (+), mild 10–30%; (+ +), moderate 30–70%; and (+ + +), strong > 70%).

## Results

### Biochemical Parameters

The blood serum testosterone, creatine, and urea levels and AST and ALT enzyme activities of the study groups are given in Table 2. The testosterone level of the CAD group was significantly lower compared to the control and SA groups (*p* < 0.0083). The testosterone level of the CAD + SA group was numerically higher than that of the CAD group (*p* > 0.05). The ALT enzyme activity of the CAD group was significantly higher compared to the control and SA groups (*p* < 0.05). The creatinine level of the CAD group was significantly higher than those of the remaining groups (*p* < 0.05).

**Table 2:** Biochemical (blood serum) parameters in the groups (*n* = 6)

Groups	Testosterone (ng/dl)	AST (U/l)	ALT (U/l)	Creatinine (mg/dl)	Urea (mg/dl)
Control	0.507 ± 0.135^a^	53.167 ± 2.197	29.500 ± 2.837^a^	0.113 ± 0.012^a^	51.850 ± 2.604
SA	0.812 ± 0.232^a^	56.833 ± 3.124	30.500 ± 1.118^a^	0.123 ± 0.006^a^	45.517 ± 1.522
CAD	0.033 ± 0.007^b^	66.667 ± 3.159	46.167 ± 6.074^b^	0.170 ± 0.009^b^	58.950 ± 4.670
CAD + SA	0.224 ± 0.131^ab^	60.833 ± 4.629	39.000 ± 2.530^ab^	0.138 ± 0.007^a^	54.217 ± 3.312
*p* value	*		**	**	

^a,b^Different letters in the same column indicate significant differences (*p < 0.0083 and **p < 0.05). *SA*, syringic acid group; *CAD*, cadmium group; *CAD* + *SA*, cadmium + syringic acid group; *ALT*, alanine aminotransferase; *AST*, aspartate transaminase

### Spermatological and Andrological Findings

**Fig. 2:**
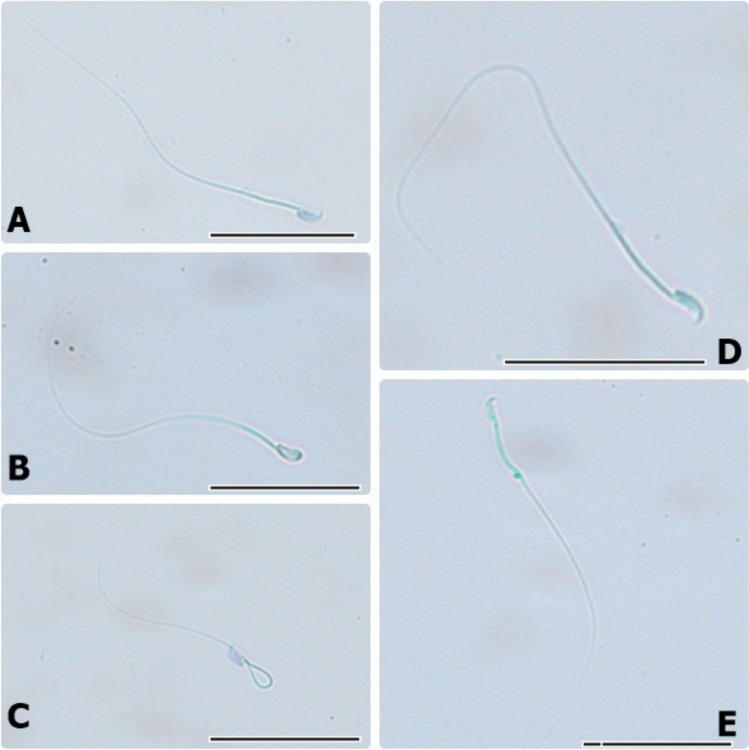
Normal and abnormal sperm images. **A** Normal sperm. **B** Head-abnormal sperm. **C** Neck-abnormal sperm. **D** Tail-abnormal sperm. **E** Cytoplasmic droplet sperm (scale bars 20 µm)

The spermatological and andrological findings of the study groups are given in Tables 3 and 4, respectively. Normal and abnormal sperm images are given in Fig. 2. No significant difference was observed between the SA and control groups in terms of sperm motility, vitality, and density (*p* > 0.05). CAD had a significant harmful effect on sperm and decreased spermatological parameters (sperm motility (%), sperm viability (%), and sperm concentration) compared to the control group (*p* < 0.0083 and *p* < 0.0001). Spermatological parameters (sperm motility (%), sperm viability (%), and sperm concentration) were better in the groups in which CAD and SA were administered together compared to the group in which only CAD was administered (*p* < 0.0001), indicating that SA reduced the toxic effect of CAD.

**Table 3:** Spermatological and andrological parameters in the groups (*n* = 6)

Groups	Sperm motility (%)	Sperm viability (%)	Sperm concentration (× 10^6^)	Right testicular weight (g)	Left testicular weight (g)
Control	83.33 ± 1.67^a^	83.67 ± 1.08^a^	9.25 ± 0.09^ab^	0.11 ± 0.02	0.11 ± 0.01
SA	81.67 ± 1.05^a^	81.5 ± 1.59^a^	9.92 ± 0.35^a^	0.12 ± 0.01	0.13 ± 0.01
CAD	7.50 ± 1.12^c^	30.50 ± 2.17^c^	2.68 ± 0.07^c^	0.053 ± 0.01	0.07 ± 0.01
CAD + SA	38.33 ± 1.05^b^	64.33 ± 2.04^b^	8.70 ± 0.20^b^	0.09 ± 0.01	0.081 ± 0.01
*p* value	*	**	**		

^a,b,c^Different letters in the same column indicate significant differences (*p < 0.0083 and **p < 0.0001). *SA*, syringic acid group; *CAD*, cadmium group; *CAD* + *SA*, cadmium + syringic acid group

**Table 4:** Abnormal sperm parameters in the groups (*n* = 6)

Groups	Head abnormal sperm (%)	Neck abnormal sperm (%)	Tail abnormal sperm (%)	Cytoplasmic droplet sperm (%)
Control	1.00 ± 0.26^a^	0.33 ± 0.28^a^	6.17 ± 0.40^b^	3.50 ± 0.43^a^
SA	1.50 ± 0.22^a^	0.50 ± 0.50^ab^	2.00 ± 0.63^a^	2.33 ± 0.33^a^
CAD	15.83 ± 1.92^c^	5.00 ± 0.77^c^	37.83 ± 2.24^d^	6.50 ± 0.43^b^
CAD + SA	7.67 ± 0.56^b^	2.67 ± 0.49^bc^	14.00 ± 0.58^c^	3.50 ± 0.42^a^
*p* value	*	*	*	**

^a,b,c,d^Different letters in the same column indicate significant differences (*p < 0.0083 and **p < 0.0001). *SA*, syringic acid group; *CAD*, cadmium group; *CAD* + *SA*, cadmium + syringic acid group

There was no significant difference between the groups in terms of testicular weight (*p* > 0.05). CAD significantly increased the rate of abnormal sperm (head abnormal sperm (%), neck abnormal sperm (%), tail abnormal sperm (%), and cytoplasmic droplet sperm (%)). SA significantly reduced this abnormality in the group CAD + SA (*p* < 0.0083 and *p* < 0.0001).

### Histopathological Findings

In the CAD group, degeneration and necrosis were widespread with 60–70% of HPFs in all cases studied. Cuboidal-shaped tubule cells and Sertoli cells were the most affected cells in necrotic seminiferous tubules. Both the nuclei and cytoplasm of these cells were severely affected by CAD. Necrotic nuclei presented with chromatin clumping, swelling of membrane integrity, disorganization of nuclear structure, and severe karyopyknosis (regular or irregular chromatin aggregation), karyolysis, and karyorrhexis. As cytoplasmic changes, cellular membrane integrity and cytoplasmic components were lost. Cytoplasms were observed as light pink in appearance. However, in the CAD + SA group, such kinds of changes were observed mildly to moderately (among 30–50% HPFs). Apoptotic seminal gland cells and Sertoli cells showed nuclear-cytoplasmic findings. The nuclei undergoing apoptosis were composed of chromatin condensation or delineation. Pyknotic uniform nuclei or crescentic condensation just beneath the nucleolemma took into attention. The cytoplasm of apoptotic cells exhibited these nuclear changes. Cytoplasmic breakdown, condensation of cellular components, shrinkage (appearing dark pink), and distinctive apoptotic bodies were observed in some cells. Scores belonging to histopathological findings have been given in Table 5.

**Table 5:** Scoring of histopathological findings in the groups (*n* = 6)

Groups	Degeneration and necrosis	Apoptosis	Regeneration	Hyperemia	Edema	Hemorrhage	Inflammatory cell infiltration
Control	-/+	-/+	+	-	-	-	-
SA	-/+	+/+ +	-/+	-	-	-	-
CAD	+ +/+ + +	+/+ +	-	-/+	-/+	-/+	-
CAD + SA	+/+ +	+/+ +	-/+	-/+	-	-	-

(-), no findings; (+), mild, in a few of microscopic fields; (+ +), in many microscopic fields; (+ + +), all microscopic field findings. *SA*, syringic acid group; *CAD*, cadmium group; *CAD* + *SA*, cadmium + syringic acid group

**Fig. 3:**
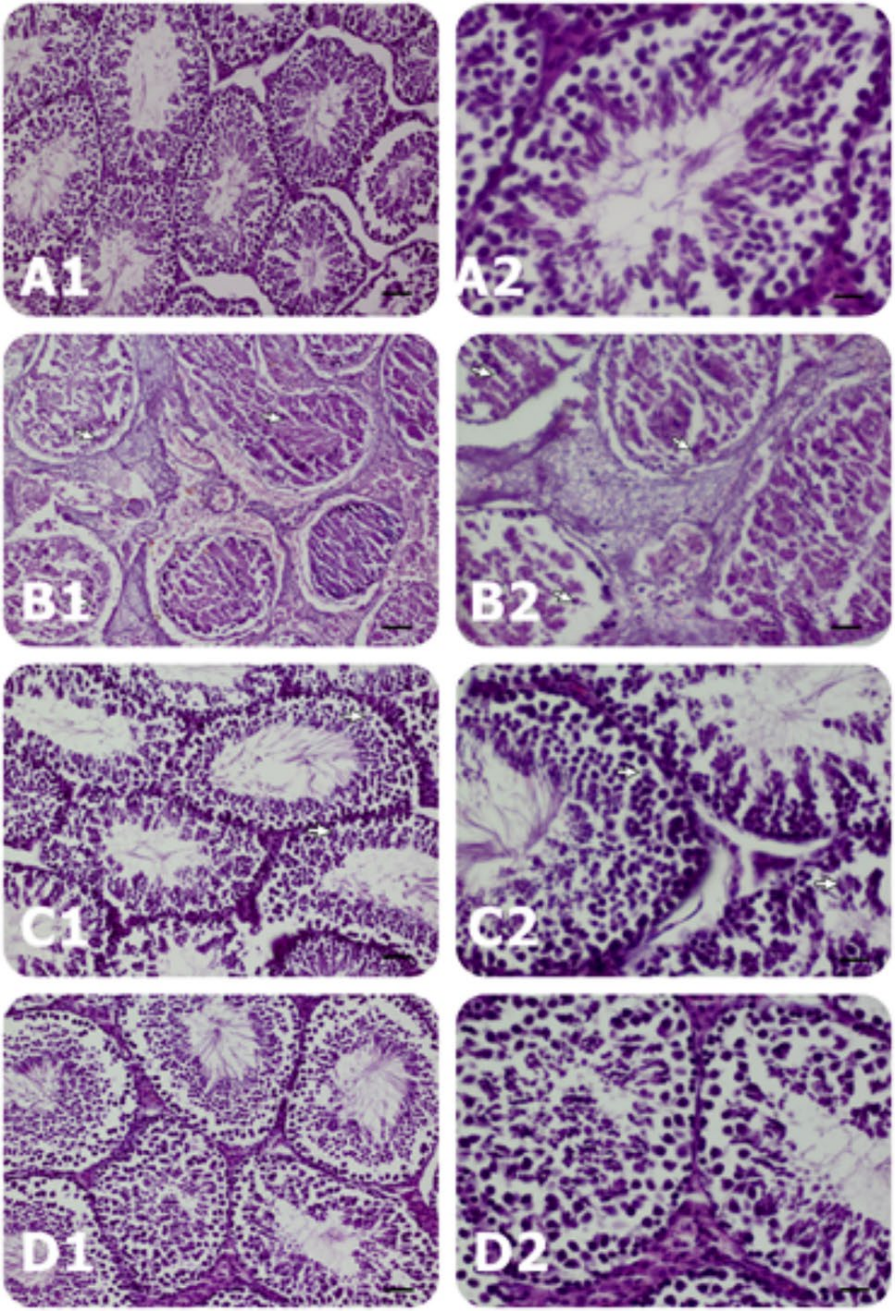
Histopathological findings in the groups. **A** Control group, **B** CAD group, **C** CAD + SA group, and **D** SA group. Control, no findings; SA, some apoptotic cells (arrows); CAD, apoptotic cells with karyopkynosis in many fields and necrotic cells of seminiferous tubules showing karyopyknotic and shrunken cytoplasm cells in some fields (arrows); and CAD + SA, apoptotic cells with karyopyknosis in a few fields in a few fields (arrows); left column bar scale (50 µm) and right column bar scale (20 µm); enlarged view of pathologic field showed within rectangle in right column; H&E staining (10 × objective, 20 × objective)

In the control group, alterations including apoptosis, degeneration, and necrosis were found in a few cell lining seminiferous tubules per microscope field. Regeneration was found to be mild, although there were no vascular changes, such as hyperemia and edema. There was also no inflammatory cell infiltration in the interstitial tissue. In the SA group, degeneration and necrosis were observed in a few cells per microscope field. However, mild to moderate apoptotic changes were detected in many cases. Regeneration findings were fewer than those in the control group. There was no inflammatory cell infiltration. In the CAD group, degeneration and necrosis were widespread in cells of all microscope fields. However, apoptotic findings were similar to the findings of the SA group. In the CAD + SA group, degeneration and necrosis were decreased when compared to the CAD group. The findings were mild to moderate in these animals. Apoptotic findings remained stable similar to the SA and CAD groups. In the CAD + SA group, regeneration findings were similar to those of the SA group. However, in this group, there were mild hyperemic vessels in some microscope fields despite the absence of severe vasculature findings. The histopathological findings in the groups are demonstrated in Fig. 3.

### Immunohistochemical Findings

The immunohistochemical findings of the study groups are given in Table 6. Positive cells containing seminiferous tubules lined by epithelial cells, Sertoli cells, and Leydig cells were light to dark brown in color in the testicles. These cells drifting into necrosis and apoptosis generally showed a positive reaction to the markers.

**Table 6:** Immunohistochemical findings in the groups (*n* = 6)

*Groups/markers*	*iNOS*	*cyclinD1*	*CB1*
Control	22.83 ± 4.08^a^	46.00 ± 10.28	36.50 ± 7.10
SA	11.50 ± 2.54^a^	36.50 ± 3.70	47.50 ± 3.82
CAD	144.17 ± 27.51^b^	30.83 ± 7.40	29.33 ± 5.75
CAD + SA	26.33 ± 6.43^a^	46.67 ± 4.68	28.50 ± 4.14
*p* value	*		

^a,b^Different letters in the same column indicate significant differences (*p < 0.0083). *SA*, syringic acid group; *CAD*, cadmium group; *CAD* + *SA*, cadmium + syringic acid group; *iNOS*, inducible nitric oxide synthase; *CB1*, cannabinoid receptor 1

#### iNOS Positivity

In the SA and CAD + SA groups, iNOS positivity had a similar distribution to the control group. However, in the CAD group, the iNOS positivity in the testicles was severely elevated (*p* < 0.0083).

#### Cyclin D1 Positivity

In the control and CAD + SA groups, cyclin D1 positivity was found to be at similar levels. In the SA group, positive reactions were less decreased when compared to these two groups. Cyclin D1 positivity decreased in the CAD group, but no statistically significant difference was observed (*p* > 0.0083).

### CB1 Positivity

**Fig. 4:**
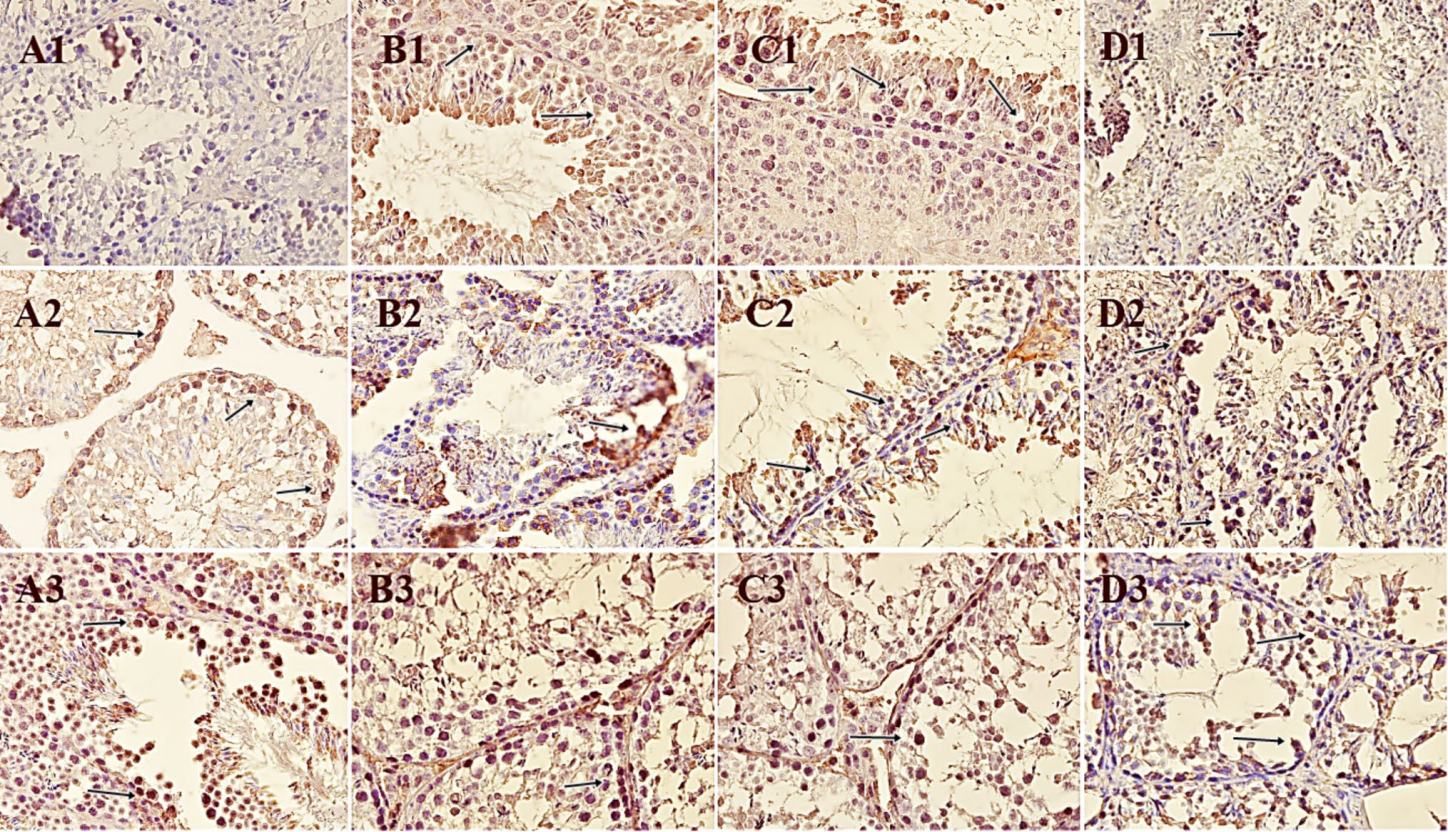
Immunopositivity of the testicles of experimental groups for different markers (arrows). **A** Control group, **B** CAD group, **C** CAD + SA group, **D** SA group, (1) iNOS, (2) cyclinD1, (3) CB1. iNOS: control showed no findings, SA showed low positivity in some apoptotic cells, CAD showed mild positivity in apoptotic and necrotic cell lining in seminiferous tubules, CAD + SA showed moderate positivity in apoptotic cells with karyopyknosis in many fields. Cyclin D1: control showed moderate positivity in cell lining seminiferous tubules; SA showed mild positivity in some cells; CAD showed low positivity in apoptotic and necrotic cell lining seminiferous tubules; CAD + SA showed moderate positivity in apoptotic cells; CB1: control showed mild positivity in cell lining seminiferous tubules; SA showed moderate positivity in some cells; CAD showed low positivity in apoptotic and necrotic cell lining seminiferous tubules; CAD + SA showed low positivity in apoptotic and degenerated cells in seminiferous tubules. Strept ABC. DAB chromogen and counterstaining with Mayer’s hematoxylin (20 × objective)

Similar to cyclin D1, the rate of CB1 positivity was similar in the control and SA groups. CB1 positivity decreased in CAD and CAD + SA groups, but no statistically significant difference was observed (*p* > 0.0083). The immunohistochemical findings of the groups are demonstrated in Fig. 4.

## Discussion

Acute or chronic CAD (Cd^+2^) toxicity causes excessive reactive oxidative substance (ROS) accumulation, resulting in lipid peroxidation in the cells of the testis [26, 28]. CAD compounds cause severe damage to the testes, as CAD can cross the blood-testis barrier. Cd^+2^ ions that cross the blood-testis barrier can penetrate into the seminiferous tubules. Ions and Cd^+2^-containing compounds disrupt cell-to-cell endothelial and epithelial junctions [29, 30]. Many studies have confirmed the relationship between testicular toxicity induced by CAD and changes in serum testosterone. A significant decrease has been found in the serum testosterone level due to testicular toxicity [31, 33]. In this study, testosterone levels decreased after CAD administration. It has been shown that SA is protective against CAD-induced testicular toxicity by increasing testosterone levels. SA has antimicrobial, anti-inflammatory, hepatoprotective effects, and its antioxidant effect in particular causes a reduction in oxidative stress within the body [13, 34]. In this study, it was proven that SA had a protective effect on spermatological parameters. CAD had a significant harmful effect on semen and decreased spermatological parameters compared to the control group. Spermatological data were better when CAD and SA were administered together compared to the group in which only CAD was administered. Thus, it was concluded that SA reduced the toxic effect of CAD. CAD has been found to be present in tissue as a result of bioaccumulation from water or diet, and the harmful effect of CAD on sperm in different species has been reported by various studies [35, 37].

In histoarchitecture, continuous CAD bioaccumulation can result in tubule distortion and disorganization. Moreover, degeneration and necrosis in Sertoli and Leydig cells (change of nuclear morphology and visible clear vacuoles), as well as germ cell lining seminiferous tubules (resulting in the complete absence of spermatozoa) can be observed. According to the literature data, Cd^+2^ toxicity generates ROS accumulation in the form of superoxide anion (O_2_^•−^), hydroxyl radicals (OH^−^), and hydrogen peroxide (H_2_O_2_); therefore, oxidative stress can develop [38, 41]. Furthermore, Cd^+2^-induces damage to DNA and other compound-making structures of cells (i.e., protein and lipid backbones) [42, 43]. In our study, we also observed these different damage mechanisms in testicular tubules. In the CAD group, degeneration and necrosis were evident across the cells in all microscope fields. In the CAD + SA group, degeneration and necrosis were decreased when compared to the CAD group. In the CAD + SA coadministered group, the findings ranged from mild to moderate degeneration in the animals. Apoptotic findings remained stable, similar to the SA and CAD groups. Regeneration findings were similar to those of the SA group.

Due to the elevated iNOS expression caused by ROS activity, Cd^+2^-induced lesions which are hemorrhage and edema as well as cellular damage, tubular disorganization, and atrophy can be more severe changed. This can reduce cellular counts per tubule and motility of sperm. Function loss in the testicles can be easily detected based on the low production of testosterone concentrations in plasma and testes [44, 45]. In the CAD group, the positivity iNOS in the testicular tubules was significantly elevated. However, in the SA and CAD + SA groups, positivity iNOS levels were similar to those in the control group. Thus, we consider that there is parallelism between positivity iNOS and histopathological alterations in the Cd^+2^-given groups. Histopathological changes, including degeneration, necrosis, and apoptosis, were diminished in the SA and CAD + SA groups. The lower of positivity iNOS, distribution of seminiferous tubules, and cellular damage were associated with the administration of SA. After continuous Cd^+2^ exposures, oxidative stress–related damage can lead to interstitial injury after tubular damage [46]. Cell lining tubules are desquamated into the lumen due to the impairment of the tubule structure and luminal contraction [47]. The tissue, including blood vessels, can be affected by ROS accumulation. The vessels are first dilated, and then congested by erythrocytes. Finally, ischemia by vasoconstriction develops due to the disintegration of the blood-testis barrier. However, fibrocytes and fibroblasts can show resistance to stress due to free radicals [29, 30, 45, 48]. Hemodynamics is affected by the disruption of junctional activity and cellular adhesion in blood vessels. Edema and free erythrocytes due to extravasation are observed in the microenvironment in the final step [29, 30, 45, 48]. In our study, the control and SA groups did not show vascular activity. However, in the CAD and CAD + SA groups, there were mild vascular changes. These changes were relatively less observed in the CAD + SA group than in the CAD group. This suggests that SA controls vascular hemodynamics and does not disturb vessel permeability. The absence of disorganization in vascular changes showed that SA can positively affect tissue health and preserve the cellular structure.

It is also known that the testis plays a potential role in preservation itself. Testicular cells undergoing oxidative stress can show several interactions to fight radical scavengers and stop free radicals. Free radicals may cause apoptosis in germ cells and impair the integrity of the blood-testis barrier. This can result in germ cell loss and vasculature changes including edema and hemorrhage [49]. It has been reported that anti-oxidants serving as potent radical scavengers may neutralize the effects of free radicals before they enter cells [49, 51].

SA is an important free radical scavenger. SA is an active compound. Radical scavenging is performed by phenolic acids, hydroxyl and methoxy groups, and an aromatic ring of benzoic acids. It has been used in biological and therapeutic applications due to its potential antioxidant, anti-microbiologic, anti-toxicant, and anti-inflammatory activities, as well as anti-proliferative and anti-neoplastic traits [13, 52, 53].

In relation to cellular regeneration, cyclins are known as regulatory subunits of the cyclin-dependent kinases machinery. Cyclins control cellular proliferation and regulate transition milestones (G1, S, G2, and M) during the cell cycle [54]. In a previous study, it was shown that the upregulation of cyclin D1 facilitated cell cycle proliferation and colony formation in a special lung epithelial cell line (CCT-LC cell line) in the presence of long Cd^+2^ exposure [55]. Therefore, in proliferating cells, cyclins interact with their specific kinases (cdk4 and/or cdk6) for cells as to be in retinoblastoma proteins (Rb) phosphorylation [54, 56].

In the literature, it has also been shown that Cdk4 is highly expressed during mitosis development in spermatogonia [57]. However, significant spermiogenesis is observed when downregulation of cyclin D1 and Cdk4 levels occurs. Both are so important in tubular cellularity and cellular differentiation or maturation under ischemia and vasoconstriction (i.e., varicoceles) in vessels [58]. In our study, we found similar cyclin D1 expression levels in the control and CAD + SA groups. However, the decreased reactions were conspicuous in the CAD group. Therefore, we consider that cyclin D1 immunopositivities may have a negative tendency in the presence of Cd^+2^-associated oxidative stress. SA may have reversed the effect of CAD and resulted in findings similar to the intact tissue of the control group animals.

## Conclusion

CAD is a potential toxicant. SA increases the rate of sperm motility, sperm viability, and sperm density caused by CAD. It also reduces abnormal sperm parameters (such as head abnormal sperm (%), neck abnormal sperm (%), tail abnormal sperm (%), cytoplasmic droplet sperm (%)) caused by CAD. Further studies should focus on investigating the protective effect of syringic acid on CAD toxicity by various mechanisms and dose–response studies.

## Data Availability

No datasets were generated or analysed during the current study.
